# The risk of colorectal cancer according to obesity status at four-year intervals: a nationwide population-based cohort study

**DOI:** 10.1038/s41598-023-36111-6

**Published:** 2023-06-01

**Authors:** Ji Yeon Seo, Eun Hyo Jin, Goh Eun Chung, Young Sun Kim, Jung Ho Bae, Jeong Yoon Yim, Kyung-Do Han, Sun Young Yang

**Affiliations:** 1 Department of Internal Medicine and Healthcare Research Institute, Healthcare System Gangnam Center, Seoul National University Hospital, 39F Gangnam Finance Center 152, Teheran-ro, Gangnam-gu, Seoul, 06236 South Korea; 2 Department of Statistics and Actuarial Science, Soongsil University, 369 Sangdo-ro, Dongjak-gu, Seoul, 06978 South Korea

**Keywords:** Cancer, Gastroenterology

## Abstract

Obesity is a risk factor for colorectal cancer. However, the effect of body weight change on colorectal cancer is uncertain. This study aimed to investigate the relationship between difference in body mass index and the risk of colorectal cancer. In this nationwide population-based cohort study, participants of the national cancer screening program in 2005 and 2009 were enrolled. Difference of body mass index was calculated from screening data from 2005 and 2009. Participants were divided into four groups according to direction of obesity status: non-obese/non-obese, non-obese/obese, obese/non-obese, and obese/obese. The effect of differences in body mass index on colorectal cancer was analyzed. Among 3,858,228 participants, 47,894 (1.24%) participants were newly diagnosed with colorectal cancer during the 9.2 years of follow-up. The incidence of colorectal cancer was higher in the obese/obese group than the non-obese/non-obese group (hazard ratio = 1.08 [1.06–1.11], *P* trend < 0.001). The men in the obese/obese group had a higher risk of colon cancer than women (hazard ratio = 1.13 [1.10–1.17] in men, and hazard ratio = 1.04 [1.01–1.18] in women, *P* = 0.001). Persistent obesity was associated with a higher risk of incidence of colorectal cancer.

## Introduction

Colorectal cancer is the third most diagnosed cancer and the second leading cause of cancer-related deaths worldwide reported in 2020^1^. Due to lifestyle changes, such as changes in diet and physical activity, incidence rates of colorectal cancer have steadily increased in many countries in Eastern Europe, South Eastern Asia, South Central Asia, and South America^2,3^. In contrast, incidence and mortality of colorectal cancer are decreasing in the United States, Australia, and European countries, including Austria, Czech Republic, and Germany. These changes are due to the adoption of healthier lifestyles and increase in colonoscopy screening programs^2,3^. In 2012, higher incidence and mortality rates of colorectal cancer were reported among men in the Republic of Korea than among men in western countries^2^, which is consistent with the increased prevalence of obesity^4^.

Obesity is defined as the excessive accumulation or abnormal distribution of body fat that affects health^4^. It is generally assessed using body mass index (BMI), and waist circumference (WC). Obesity is well known as a risk factor of various cancers, including breast, prostate, kidney, esophageal, endometrial, and colorectal cancer^5,6^. Adipose tissue works as an endocrine organ that can cause abnormal inflammation and contribute to systemic metabolic diseases, such as hypertension, diabetes mellitus, dyslipidemia, and coronary heart disease^7^. Weight change over time might reflect age-related metabolic changes. From a public health perspective, the effect of weight changes over a period of time on the risk of cancer should be elucidated^8^.

Among various factors, the relationship between obesity and colorectal cancer has been well established^3,9^. Previous studies have shown that the relative risk of developing colorectal cancer among men with obesity ranges from 1.37 to 1.95^10,11^ and remains unclear in women^10–12^. Additionally, obesity has been suggested to have an association with colorectal cancer-related mortality^13^ or recurrence^14^.

Weight changes occur in different individuals at different times of life. Although lifestyle modifications may be needed for weight changes, knowledge on how weight changes affect risk of colorectal cancer is necessary before recommending lifestyle modifications. Several studies have demonstrated that weight change in men is associated with colorectal cancer but not in women^15–18^. However, the results of these studies vary, and the association between weight change and risk of colorectal cancer remains debatable^19–21^. Few studies have evaluated the effect of weight change on colorectal cancer by comparing age and sex^19^. Hence, this large cohort study aimed to evaluate the relationship between obesity status and incidence of colorectal cancer according to subgroups such as age and sex among Koreans who participated in the national health screening program.

## Results

### Demographic and clinical characteristics

A total of 3,858,228 individuals who participated in the national screening programs in 2005 and 2009 were included in this study. Among them, 47,894 (1.24%) participants were diagnosed with colorectal cancer. Baseline characteristics of participants are shown in Table 1. The mean age of participants with colorectal cancer was higher than that of participants without colorectal cancer (59.0 ± 11.6 *vs* 49.0 ± 12.9 years, *P* < 0.001). The number of men with colorectal cancer was higher than that of women (64.2% *vs* 35.8%). Significant between-group differences in economic status, smoking, alcohol intake, regular physical activity, BMI, WC, history of diabetes, hypertension, and dyslipidemia were present (all *P* < 0.001). The initial mean BMI was 24.1 ± 3.0 in patients with colorectal cancer and 23.7 ± 3.1 in controls (P < 0.001). Final mean BMI level was higher among patients with colorectal cancer (24.1 ± 3.0 vs. 23.8 ± 3.1, P < 0.001). Overall, 40.0% of participants were underweight or normal (BMI < 23 kg/m^2^), 26.2% were overweight (BMI, 23.0–24.9 kg/m^2^), and 33.8% were obese (BMI ≥ 25.0 kg/m^2^). Mean follow-up durations for colorectal cancer, colon cancer, and rectal cancer were 9.18 ± 1.1, 9.18 ± 1.1, and 9.21 ± 1.1 years, respectively.

**Table 1:** Comparison of baseline characteristics according to colorectal, colon, and rectal cancer.

	Colorectal cancer			Colon cancer			Rectal cancer		
	No	Yes	*P* value	No	Yes	*P* value	No	Yes	*P* value
N (%)	3,810,334 (98.8)	47,894 (1.24)		3,817,621 (99.0)	40,607 (1.1)		3,844,434 (99.6)	13,794 (0.4)	
Age (year)	49.0 ± 12.9	59.0 ± 11.6	< 0.001	49.0 ± 12.9	59.3 ± 11.4	< 0.001	49.1 ± 13.0	58.4 ± 11.9	< 0.001
Sex			< 0.001			< 0.001			< 0.001
Men	2,321,227 (60.9)	30,728 (64.2)		2,326,445 (60.9)	25,510 (62.8)		2,342,245 (60.9)	9710 (70.4)	
Women	1,489,107 (39.1)	17,166 (35.8)		1,491,176 (39.1)	15,097 (37.2)		1,502,189 (39.1)	4084 (29.6)	
Economic status (Low)	556,645 (14.6)	9,164 (19.1)	< 0.001	557,980 (14.6)	7829 (19.3)	< 0.001	563,229 (14.7)	2580 (18.7)	< 0.001
Smoking			< 0.001			< 0.001			< 0.001
Non-smoker	2,140,588 (56.2)	26,531 (55.4)		2,144,161 (56.2)	22,958 (56.5)		2,160,140 (56.2)	6979 (50.6)	
Ex-smoker	668,453 (17.5)	9,783 (20.4)		670,043 (17.6)	8193 (20.2)		675,299 (17.6)	2937 (21.3)	
Current smoker	1,001,293 (26.3)	11,580 (24.2)		1,003,417 (26.3)	9456 (23.3)		1,008,995 (26.3)	3878 (28.1)	
Alcohol intake			< 0.001			< 0.001			< 0.001
None	1,899,249 (49.8)	25,391 (53.0)		1,902,752 (49.8)	21,888 (53.9)		1,917,835 (49.9)	6805 (49.3)	
Mild (< 30 g/day)	1,602,219 (42.1)	18,004 (37.6)		1,605,239 (42.1)	14,984 (36.9)		1,614,716 (42)	5507 (39.9)	
Heavy (≥ 30 g/day)	308,866 (8.1)	4,499 (9.4)		309,630 (8.1)	3735 (9.2)		311,883 (8.1)	1482 (10.7)	
Regular physical activity	754,268 (19.8)	10,514 (22.0)	< 0.001	755,814 (19.8)	8968 (22.1)	< 0.001	761,816 (19.8)	2966 (21.5)	< 0.001
BMI (previous, kg/m^2^)	23.7 ± 3.1	24.1 ± 3.0	< 0.001	23.7 ± 3.1	24.1 ± 3.0	< 0.001	23.7 ± 3.1	24.0 ± 3.0	< 0.001
BMI (post, kg/m^2^)	23.8 ± 3.1	24.1 ± 3.0	< 0.001	23.8 ± 3.1	24.2 ± 3.0	< 0.001	23.8 ± 3.1	24.1 ± 3.0	< 0.001
BMI category (kg/m^2^)			< 0.001			< 0.001			< 0.001
< 23	1,525,918 (40.0)	16,996 (35.5)		1,528,612 (40.0)	14,302 (35.2)		1,537,888 (40.0)	5026 (36.4)	
23–25	999,641 (26.2)	13,095 (27.3)		1,001,614 (26.2)	11,122 (27.4)		1,009,011 (26.2)	3725 (27.0)	
≥ 25	1,284,775 (33.7)	17,803 (37.2)		1,287,395 (33.7)	15,183 (37.4)		1,297,535 (33.8)	5043 (36.6)	
WC (cm)	81.0 ± 8.7	83.2 ± 8.4	< 0.001	81.0 ± 8.7	83.2 ± 8.5	< 0.001	81.0 ± 8.7	83.4 ± 8.2	< 0.001
Diabetes mellitus	336,142 (8.8)	7,546 (15.8)	< 0.001	337,241 (8.8)	6447 (15.9)	< 0.001	341,582 (8.9)	2106 (15.3)	< 0.001
Hypertension	1,082,840 (28.4)	21,613 (45.1)	< 0.001	1,085,928 (28.5)	18,525 (45.6)	< 0.001	1,098,417 (28.6)	6036 (43.8)	< 0.001
Dyslipidemia	712,540 (18.7)	11,438 (23.9)	< 0.001	714,188 (18.7)	9790 (24.1)	< 0.001	720,794 (18.8)	3184 (23.1)	< 0.001

Values are expressed as mean ± standard deviation or frequencies (percentages).

BMI, body mass index; WC, waist circumference.

### Risk of colorectal cancer according to obesity status

The risk of colorectal cancer was assessed according to three BMI categories: BMI < 23 kg/m^2^, BMI, 23.0–24.9 kg/m^2^, and BMI ≥ 25.0 kg/m^2^ (Table 2). The risk of colorectal cancer and colon cancer increased as BMI increased. In contrast, no significant increase in risk of rectal cancer was observed among participants with obesity. The association between obesity status and incidence of colorectal cancer was evaluated (Table 3). Hazard ratios (HR) was adjusted for age, sex, smoking, alcohol intake, regular physical activity, economic status, diabetes mellitus, hypertension, and dyslipidemia. Based on the BMI of 25 kg/m^2^, the effect of difference in BMI on the incidence of colorectal cancer below and above 25 kg/m^2^ was evaluated. Participants were divided into four groups, as previously described. The risk of colorectal cancer among participants with body weight change was not significantly different from that among participants in the reference group (non-obese/non-obese group) (HR, 1.02; 95% CI, 0.98–1.06 in the non-obese/obese group and HR, 1.04; 95% CI, 0.99–1.07 in the obese/non-obese group). However, the incidence of colorectal cancer increased significantly in the obese/obese group (HR, 1.08; 95% CI, 1.06–1.11 in the obese-to-obese group, *P* trend < 0.001) (Table 3).

**Table 2:** Risk of colorectal cancer according to body mass index.

	BMI (kg/m^2^)	N	Cancer (%)	Incidence rate*	HR (95% CI)
Colorectal cancer	< 23	1,542,914	16,996 (1.10)	1.20	1 (Reference)
	< 25	1,012,736	13,095 (1.29)	1.41	1.03 (1.01–1.05)
	≥ 25	1,302,578	17,803 (1.37)	1.49	1.08 (1.06–1.10)
	*P* value				< 0.0001
Colon cancer	< 23	1,542,914	14,302 (0.93)	1.01	1 (Reference)
	< 25	1,012,736	11,122 (1.10)	1.19	1.04 (1.01–1.07)
	≥ 25	1,302,578	15,183 (1.17)	1.27	1.10 (1.07–1.12)
	*P* value				< 0.0001
Rectal cancer	< 23	1,542,914	5026 (0.33)	0.35	1 (Reference)
	< 25	1,012,736	3725 (0.37)	0.40	0.99 (0.95–1.03)
	≥ 25	1,302,578	5043 (0.39)	0.42	1.04 (1–1.08)
	*P* value				0.0343

Hazard ratio (95% CI) was adjusted for age, sex, smoking, alcohol intake, regular physical activity, economic status, diabetes mellitus, hypertension, and dyslipidemia.

Incidence rate* is defined as cancer cases per 1000 person-years.

BMI, body mass index; HR, hazard ratio; CI, confidence interval.

**Table 3:** Risk of colorectal cancer according to weight differences.

	BMI (kg/cm^2^)		N	Cancer (%)	Incidence rate*	HR (95% CI)
	Previous	Post				
Colorectal cancer	< 25	< 25	2,349,662	27,045 (1.15)	1.25	1 (Reference)
		≥ 25	283,415	3311 (1.17)	1.27	1.02 (0.98–1.06)
	≥ 25	< 25	205,988	3046 (1.48)	1.62	1.04 (0.99–1.07)
		≥ 25	1,019,163	14,492 (1.42)	1.55	1.08 (1.06–1.11)
	*P* value					< 0.001
Colon cancer	< 25	< 25	2,349,662	22,845 (0.97)	1.06	1 (Reference)
		≥ 25	283,415	2777 (0.98)	1.06	1.01 (0.97–1.05)
	≥ 25	< 25	205,988	2579 (1.25)	1.37	1.03 (0.99–1.08)
		≥ 25	1,019,163	12,406 (1.22)	1.32	1.10 (1.07–1.12)
	*P* value					< 0.001
Rectal cancer	< 25	< 25	2,349,662	7897 (0.34)	0.37	1 (Reference)
		≥ 25	283,415	947 (0.33)	0.36	1.00 (0.94–1.07)
	≥ 25	< 25	205,988	854 (0.41)	0.45	1.01 (0.94–1.08)
		≥ 25	1,019,163	4096 (0.40)	0.44	1.06 (1.02–1.10)
	*P* value					< 0.001

Hazard ratio (95% CI) was adjusted for age, sex, smoking, alcohol intake, regular physical activity, economic status, diabetes mellitus, hypertension, and dyslipidemia.

Incidence rate* is defined as cancer cases per 1000 person-years, and not adjusted.

BMI, body mass index; HR, hazard ratio; CI, confidence interval.

This trend was similar in the subgroup analysis of both colon and rectal cancer. Risks of colon and rectal cancer were higher in the obese/obese group than in the reference group (HR, 1.10; 95% CI, 1.07–1.12; *P* trend < 0.001 in colon cancer and HR, 1.06; 95% CI, 1.02–1.10; *P* trend < 0.001 in rectal cancer, respectively) (Table 3).

### Cumulative obesity burden and risk of colorectal cancer

**Figure 1:**
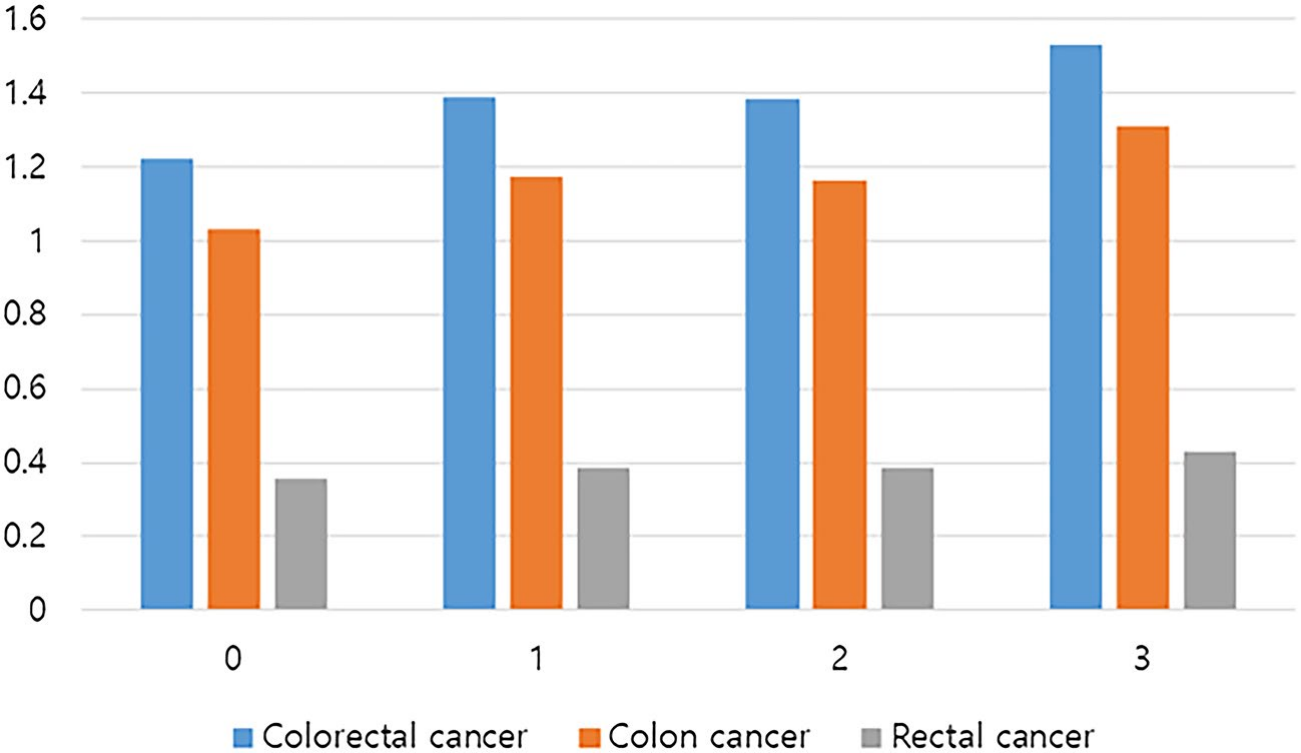
Incidence of colorectal cancer according to the cumulative obesity burden.

In Korea, routine health check-up is recommended every two years using the National Health Insurance Service (NHIS). Therefore, some participants underwent health check-up at least once during the period between 2005 and 2009. Among those who had been screened in 2005, 2007, and 2009, cumulative obesity burden was assessed. Cumulative obesity burden was defined as the cumulative number of confirmed obesity (BMI ≥ 25 kg/cm^2^) during three consecutive health check-ups and estimated from 0 to 3. Risk of colorectal cancer was analyzed according to cumulative obesity burden (Table 4 and Fig. 1). The incidence of colorectal cancer increased with cumulative obesity burden. Cumulative obesity burden of 3 showed a more significant increase in risk of colorectal cancer than the cumulative obesity burden of 0 (HR, 1.10; 95% CI, 1.07–1.13; *P* trend < 0.001). Cumulative obesity burden of 1 or 2 was not significantly different from that of non-obese subjects. A similar trend was observed in the subgroup analysis of colon cancer and rectal cancer (HR, 1.12; 95% CI, 1.09–1.15; *P* trend < 0.001 and HR, 1.07; 95% CI, 1.02–1.12; *P* trend = 0.016, respectively) (Table 4).

**Table 4:** Risk of colorectal cancer according to cumulative obesity burden.

	Cumulative obesity burden	N	EVENT	Incidence rate*	HR (95% CI)
Colorectal cancer	0	1,806,795	20,314	1.22	1 (Reference)
	1	268,236	3420	1.39	1.02 (0.99–1.06)
	2	253,839	3228	1.38	1.00 (0.97–1.04)
	3	741,939	10,459	1.53	1.10 (1.07–1.13)
	*P* value				< 0.001
Colon cancer	0	1,806,795	17,149	1.03	1 (Reference)
	1	268,236	2899	1.18	1.03 (0.99–1.07)
	2	253,839	2721	1.17	0.99 (0.96–1.04)
	3	741,939	8968	1.31	1.12 (1.09–1.15)
	*P* value				< 0.001
Rectal cancer	0	1,806,795	5935	0.36	1 (Reference)
	1	268,236	956	0.39	0.99 (0.92–1.06)
	2	253,839	907	0.39	0.97 (0.91–1.04)
	3	741,939	2934	0.43	1.07 (1.02–1.12)
	*P* value				0.016

Incidence rate* is defined as cancer cases per 1000 person-years.

Cumulative obesity burden, number of times an individual is diagnosed with obesity (BMI ≥ 25 kg/m^2^) among 3 screening programs in 2005, 2007, and 2009; HR, hazard ratio; CI, confidence interval.

### Effects of difference in BMI on colorectal cancer according to age, sex, and abdominal obesity

The colorectal cancer risk associated with difference in BMI was compared based on age, sex, and abdominal obesity (Table 5). Colorectal cancer was significantly associated with the obese/obese group only among participants aged ≥ 50 years (HR, 1.09; 95% CI, 1.07–1.12; *P* for interaction = 0.004). A similar trend was observed for colon cancer. (HR, 1.12; 95% CI, 1.09–1.14; *P* for interaction < 0.001). Based on sex, men in the obese/non-obese and obese/obese groups had an increased risk of colorectal cancer (HR, 1.06; 95% CI, 1.01–1.11 and HR, 1.11; 95% CI, 1.08–1.14, respectively). However, for women, this observation was not significant (*P* for interaction = 0.008). Interestingly, the risk of colon cancer differed by sex (*P* for interaction = 0.001), but this difference was not significant in the case of rectal cancer (*P* for interaction = 0.606). According to the presence of abdominal obesity (waist circumference ≥ 90 cm in men and ≥ 85 cm in women), colorectal cancer was significantly increased in individuals with abdominal obesity in the obese/obese group (HR, 1.06; 95% CI, 1.01–1.11). However, no significant difference existed among groups according to abdominal obesity (*P* for interaction = 0.340).

**Table 5:** Subgroup analysis of risk of colorectal cancer according to age, sex, and abdominal obesity.

	BMI (kg/m^2^)			Colorectal cancer			Colon cancer			Rectal cancer		
	Previous	Post	Total N	Cancer	Incidence rate*	HR (95% CI)	Cancer	Incidence rate*	HR (95% CI)	Cancer	Incidence rate*	HR (95% CI)
Age < 50	< 25	< 25	1,302,595	6084	0.50	1 (Reference)	4911	0.40	1 (Reference)	1,891	0.16	1 (Reference)
		≥ 25	159,737	762	0.51	0.96 (0.89–1.04)	605	0.41	0.95 (0.88–1.04)	247	0.17	0.98 (0.86–1.12)
	≥ 25	< 25	87,842	502	0.62	1.04 (0.95–1.14)	400	0.49	1.03 (0.93–1.14)	155	0.19	1.01 (0.86–1.19)
		≥ 25	490,282	2731	0.60	1.00 (0.96–1.05)	2146	0.47	0.99 (0.94–1.04)	935	0.21	1.07 (0.98–1.15)
Age ≥ 50	< 25	< 25	1,047,067	20,961	2.22	1 (Reference)	17,934	1.90	1 (Reference)	6006	0.63	1 (Reference)
		≥ 25	123,678	2549	2.27	1.03 (0.99–1.08)	2172	1.93	1.03 (0.98–1.08)	700	0.62	1.01 (0.93–1.09)
	≥ 25	< 25	118,146	2544	2.40	1.03 (0.99–1.07)	2179	2.05	1.03 (0.99–1.08)	699	0.65	1.00 (0.93–1.08)
		≥ 25	528,881	11,761	2.44	1.09 (1.07–1.12)	10,260	2.13	1.12 (1.09–1.14)	3161	0.65	1.05 (1.01–1.10)
*P* for interaction						0.004			< 0.001			0.966
Men	< 25	< 25	1,346,095	17,211	1.40	1 (Reference)	14,207	1.16	1 (Reference)	5578	0.45	1 (Reference)
		≥ 25	189,176	2161	1.24	1.03 (0.98–1.07)	1784	1.03	1.03 (0.98–1.08)	669	0.38	0.99 (0.911–1.07)
	≥ 25	< 25	128,746	1948	1.67	1.06 (1.01–1.11)	1618	1.38	1.06 (1.01–1.12)	577	0.49	0.98 (0.90–1.07)
		≥ 25	687,938	9408	1.49	1.11 (1.08–1.14)	7901	1.25	1.13 (1.10–1.17)	2886	0.46	1.06 (1.01–1.11)
Women	< 25	< 25	1,003,567	9834	1.06	1 (Reference)	8638	0.93	1 (Reference)	2319	0.25	1 (Reference)
		≥ 25	94,239	1150	1.32	1.01 (0.95–1.07)	993	1.14	0.98 (0.92–1.05)	278	0.32	1.04 (0.92–1.17)
	≥ 25	< 25	77,242	1098	1.55	0.99 (0.93–1.06)	961	1.36	0.98 (0.92–1.05)	277	0.39	1.07 (0.95,1.22)
		≥ 25	331,225	5084	1.66	1.04 (1–1.07)	4505	1.47	1.04 (1.01–1.08)	1210	0.40	1.06 (0.99–1.14)
*P* for interaction						0.008			0.001			0.606
Abdominal obesity (−)	< 25	< 25	2,251,569	25,046	1.21	1 (Reference)	21,135	1.02	1 (Reference)	7311	0.35	1 (Reference)
		≥ 25	199,762	2012	1.09	0.99 (0.95–1.04)	1685	0.91	0.99 (0.94–1.04)	567	0.31	0.96 (0.88–1.04)
	≥ 25	< 25	177,219	2414	1.49	1.04 (0.99–1.08)	2034	1.25	1.03 (0.99–1.08)	680	0.42	1.01(0.93–1.09)
		≥ 25	454,808	5292	1.26	1.02 (0.99–1.06)	4480	1.07	1.03 (0.99–1.07)	1552	0.37	1.03 (0.97–1.09)
Abdominal obesity (+)	< 25	< 25	98,093	1999	2.27	1 (Reference)	1710	1.94	1 (Reference)	586	0.66	1 (Reference)
		≥ 25	83,653	1299	1.70	1.01 (0.94–1.08)	1092	1.43	1.00 (0.93–1.08)	380	0.50	0.99 (0.87–1.13)
	≥ 25	< 25	28,769	632	2.47	0.99 (0.91–1.08)	545	2.13	1.00 (0.91–1.10)	174	0.68	0.94 (0.79–1.11)
		≥ 25	564,355	9200	1.78	1.06 (1.01–1.11)	7926	1.53	1.08 (1.02–1.14)	2544	0.49	0.99 (0.90–1.08)
*P* for interaction						0.340			0.308			0.612

Incidence rate* is defined as cancer cases per 1000 person-years.

BMI, body mass index; HR, hazard ratio; CI, confidence interval.

Hazard ratio (95% CI) was adjusted for smoking, alcohol intake, regular physical activity, economic status, diabetes mellitus, hypertension, and dyslipidemia. Abdominal obesity was defined as waist circumference ≥ 90 cm in men and ≥ 85 cm in women.

## Discussion

In this large nationwide population-based cohort study, the association between obesity status at a four-year intervals and risk of colorectal cancer was analyzed. The incidence of colorectal cancer was higher in the obese/obese group than in the non-obese/non-obese group. This trend was more apparent in men, especially for colon cancer. The obese/obese group in older age over 50 was significantly associated with colorectal cancer but not at a younger age.

With respect to the study design, the time interval between body weight measurements was 4 years, which was shorter than that of previous studies that compared body weight during early adulthood^15,17,19^. Therefore, this study focused more on short-term weight difference or fluctuation of body weight rather than long-term weight change. After analysis of data on subjects who participated in health check-ups every 2 years, persistent obesity apparently increased the risk of colorectal cancer. On the contrary, participants whose weights were changed from obese to non-obese and non-obese to obese did not show significant increase of the risk of colorectal cancer compared to non-obese/non-obese participants. And, when cumulative obesity burden is one or two out of three, the incidence of colorectal cancer was slightly higher than in the cumulative obesity burden of zero (Table 4). Therefore, if the follow-up period was longer, statistically significant results would have been obtained in obese to non-obese and non-obese to obese groups. However, our study had a short observation period, making it difficult to accurately assess the risk of weight change. Further research is needed to determine whether weight loss in obesity could normalize the risk of colorectal cancer.

In addition, whether weight loss decreases the risk of colorectal cancer than stable weight is not clear yet. Comparing the gaps of body weights at 2 different points, two studies have shown that weight loss did not have significant protective effect on colorectal cancer compared to stable weight^17,20^. Only one study showed decreased risk of colon cancer in men after weight loss^16^. Weight loss is one of the important features of colorectal cancer and there seem to be many things to consider, such as the possibility of intentional and unintentional weight loss, and weight loss due to other causes. The effect of weight loss on colorectal cancer needs further investigation.

Excluding hereditary factors and health conditions, such as inflammatory bowel disease, colorectal cancer is well-known to be associated with lifestyle and dietary factors. Up to 45–47% of colorectal cancer cases are estimated to be attributable to lifestyle and dietary factors, which are modifiable risk factors^3^. Evidence from previous studies shows that the relative risk of developing colorectal cancer was highest with alcohol consumption, smoking, processed meat consumption, and obesity, in that order^3^. In this study, the relative risk of colorectal cancer in participants with obesity was 1.05 (95% CI, 1.03–1.07)^3^. To compare the effects of other factors, the risk of colorectal cancer was analyzed by adjusting for age and sex only. A higher risk was initially observed than when smoking, alcohol consumption, exercise, economic status, and metabolic diseases were adjusted (HR, 1.12; 95% CI, 1.09–1.14 and HR, 1.08; 95% CI, 1.06–1.11, respectively). Inferring from these results, multiple factors interact with colorectal cancer. To prevent colorectal cancer, weight loss, metabolic disease management, and comprehensive lifestyle changes among individuals with obesity are necessary. Particularly in men, the effect of smoking cessation and alcohol consumption reduction would be more profound.

In this study, the subgroup analysis based on age revealed that the obese/obese group was significantly associated with colorectal cancer in the older age group (> 50 years) than in the younger age group. Focusing on the number of subjects, most subjects did not experience significant weight change. In the old age group, 89.4% and 81.7% of participants with and without obesity, respectively, experienced no alterations in BMI status. According to a previous study, weight gain does not usually occur in old age. Rather, obesity in old age is mainly due to ageing of individuals with obesity during adulthood^22^. Considering our results on cumulative obesity burden, long-term obesity is expected to play a role in colorectal cancer. Not only the incidence but also the mortality of colorectal cancer was reported to be associated with a higher BMI among elderly participants^23^. Since the observation period in this study was as short as 4 years, further studies are needed to evaluate the effect of duration of obesity.

Previous studies^15–18,20,21^, except for one European prospective cohort study,^19^ reported no association between weight change and colorectal cancer, when men and women were combined. The incidence of colorectal cancer was reported to be significantly higher in men with obesity than in women with obesity^15^. This sex difference might be explained difference in prevalence of colorectal cancer, difference in incidence of metabolic syndrome, or the protective effect of estrogen in women^10,11,24^. The effect of weight change on colorectal cancer in men was clear, but not in women^15–19^. Only two studies could not prove the significant association between weight change and colorectal cancer in men^20,21^. In our study, although the risk of obesity was higher in men, both men and women showed increased risk of colon cancer in the obese/obese group. The different results could be due to difference in race, weight comparison point, and comparison tools, such as body weight, BMI, and WC. Since estrogen is an important difference between men and women, pre- and post-menopausal women were compared by stratifying women by age. We used a cut-off of 50 years, which is the mean age of menopause in Korean women. Yet, no statistical difference was observed between old and young women (*P* for interaction = 0591, data not shown). Existing studies have reported conflicting results on the relationship between menopause status and colorectal cancer^25,26^. Further research is needed to clarify the effect of menopause on obesity and colorectal cancer.

This study had several limitations. First, due to the retrospective study design, body weights at the time of screening were used. Since the weight difference between the two time points was compared, the trend of change in body weight might not be reflected. Furthermore, the four-year interval used for comparing body weight change was deemed insufficient in assessing changes in obesity status. We consider this to be a crucial reason why there was no significant findings in the groups with changes in obesity status such as obese to non-obese and non-obese to obese. Second, only Asians were included in this study, and according to the recommendations of WHO, based on the Asia–Pacific perspective, BMI ≥ 25 kg/m^2^ was defined as obesity. This is different from the general World Health Organization (WHO) guidelines that considers BMI ≥ 30 kg/m^2^ as obesity. Therefore, findings from this study are limited in application to other races. Third, since data were collected from mass screening programs, BMI was used to compare obesity status. Visceral abdominal fat and WC are suggested to be better predictors of colon cancer^25^.

Despite these limitations, the significance of this study is emphasized by its large cohort size, which is the largest cohort study that has evaluated the association between obesity status and risk of colorectal cancer. Furthermore, a nationwide population-based cohort from the national health insurance database was adopted. The data from the database were standardized. For example, licensed medical staff recorded BMI data, and no information provided by recall was used. The duration of difference in BMI was the same for all participants and it could reflect the rate of weight change.

## Conclusion

In conclusion, persistent obesity (BMI ≥ 25 kg/m^2^) was significantly associated with colorectal cancer in both men and women. Hence, prevention of persistent obesity and avoiding weight gain would help prevent colorectal cancer in both men and women.

## Material and methods

### Data source

This nationwide population-based cohort study was conducted using the National Health Insurance Corporation (NHIC) database. NHIC collects data from all Koreans enrolled in the NHIS, which is a government agency that provides mandatory universal coverage to 97% of the Korean population^27^. This database includes all healthcare-related information, including diagnosis, treatment, and prescriptions, recorded for reimbursement by the Health Insurance and Review Agency. The NHIS conducts biennial health screening programs for all Koreans aged above 40 years and all employees regardless of age^28^. Therefore, the NHIC database includes not only highly qualified data, including demographic data, anthropometric measurements, laboratory blood and urine test results, findings from questionnaires on lifestyle, and medical records, but also trends of change of these variables.

### Study design

**Figure 2:**
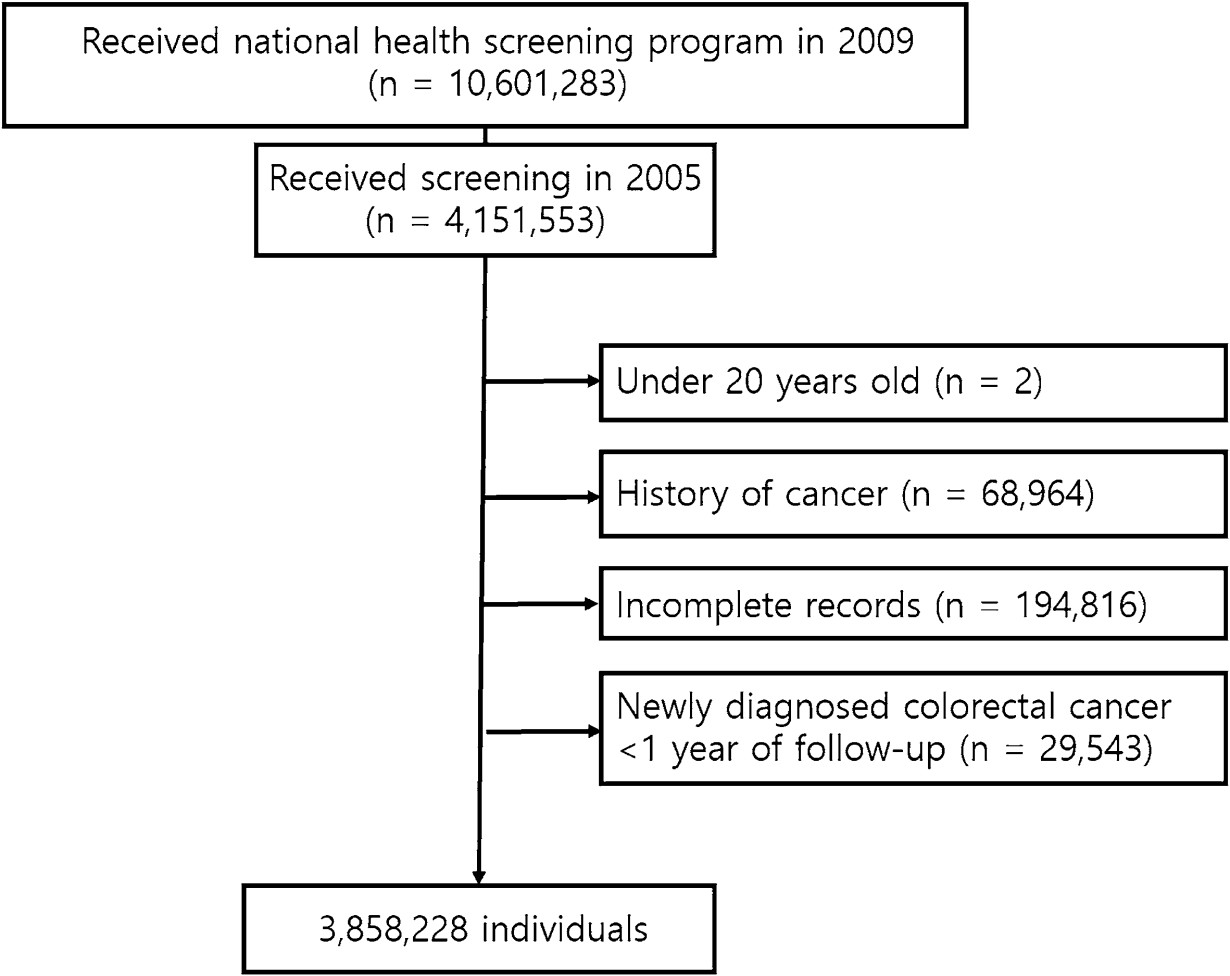
Flow chart of enrollment process in this study.

Among 10,601,283 individuals who participated in the national health screening program in 2009, 4,151,553 individuals who were previously screened in 2005 were selected. Individuals under the age of 20 years (n = 2), with a history of any type of cancer before the examination (n = 68,964), with incomplete medical information (n = 194,816), with diagnosis of colorectal cancer within a year of follow-up (n = 29,543) were excluded. Finally, 3,858,228 participants were recruited. The flow chart of participant selection is displayed in Fig. 2.

This study was approved by the Ethics Committee of Seoul National University Hospital (IRB No. H-2201-011-1286); it conformed to the ethical guidelines of the World Medical Association’s Declaration of Helsinki. The requirement for informed consent from individual participants was waived by Ethics Committee of Seoul National University Hospital, because this study used de-identified collected data. All authors had access to the study data and reviewed and approved the final manuscript.

### Variables

Study data consisted of medical information based on a self-administered questionnaire and anthropometric and laboratory measurements. Height and body weight were measured using a digital scale recorded by medical staff. The BMI was calculated as a ratio of weight to height^2^ (kg/m^2^). BMI was divided into five categories based on the guidelines of the Korean Society for the Study of Obesity (KSSO) and recommendations of the WHO based on the Asia–Pacific perspective^29^: underweight (< 18.5 kg/m^2^), normal-weight (18.5–22.9 kg/m^2^), overweight (23.0–24.9 kg/m^2^), obesity (25.0–29.9 kg/m^2^), and severe obesity (≥ 30.0 kg/m^2^)^29^. According to the WHO Asia–Pacific definition, BMI ≥ 25 kg/m^2^ is defined as obese and BMI < 25 kg/m^2^ is defined as non-obese^29^.

Difference of body weight was defined as the difference between the weights of the participants measured at national health examination programs in 2005 and 2009^30^. Participants were divided into four groups according to weight differences: non-obese/non-obese (BMI < 25 to < 25 kg/m^2^), non-obese/obese (BMI < 25 to ≥ 25 kg/m^2^), obese/non-obese (BMI ≥ 25 to < 25 kg/m^2^), and obese/obese (BMI ≥ 25 to ≥ 25 kg/m^2^). The non-obese/non-obese group was designated as the reference group. Based on standardized self-reporting questionnaires^28^, smoking status was categorized as non-smoker, ex-smoker, and current smoker. Alcohol intake was categorized into three groups: non-drinkers, mild (< 30 g of alcohol/day) drinkers, and heavy (≥ 30 g of alcohol/day) drinkers. Regular physical activity was defined as 30 min of moderate physical activity for more than 5 days per week. Hypertension was defined as systolic blood pressure ≥ 140 mmHg, diastolic blood pressure ≥ 90 mmHg, or a history of receiving antihypertensive medications. Diabetes was defined as fasting glucose levels ≥ 126 mg/dL, glycated hemoglobin level (HbA1c) ≥ 6.5%, or a history of receiving glucose-lowering agents.

### Outcomes

The primary outcome of this study was new colorectal cancer cases, defined as having the International Classification of Diseases, Tenth Revision (ICD-10) code of C18–C20 diagnosed either in inpatient or outpatient settings. Colon and rectal cancer were defined as C18.0 and C20.0, respectively. Licensed physicians registered the diagnosis of cancer cases according to the requirements of the National Cancer Registration Project, which is mandatory when cancer is diagnosed. The incidence of colorectal cancer was calculated as a ratio of the number of events to person-time at risk. Participants were followed until the diagnosis of colorectal cancer, death, or the last day of the study period (December 2019).

### Statistical analysis

Variables were expressed as means ± standard deviation or frequencies (percentages). To determine the risk of colorectal cancer, Cox proportional hazard model was applied; the model adjusted for age, sex, smoking, alcohol intake, regular physical activity, economic status, diabetes mellitus, hypertension, and dyslipidemia. HR and 95% confidence intervals (CIs) of colorectal cancer were calculated according to the short-term change in BMI and cumulative obesity burden (number of confirmed obesity, BMI ≥ 25 kg/m^2^, during three consecutive check-ups). The non-obese/non-obese group was considered as the reference group (BMI < 25 to BMI < 25 kg/m^2^). Variables with *P*-values < 0.05 were considered statistically significant. Statistical analyses were performed using SAS version 9.4 (SAS Institute Inc., Cary, NC, USA).

### Ethics declarations and Informed consent statement

This study was approved by the Ethics Committee of Seoul National University Hospital (IRB No. H-2201-011-1286); it conformed to the ethical guidelines of the World Medical Association’s Declaration of Helsinki. The requirement for informed consent from individual participants was waived, because this study used de-identified collected data. All authors had access to the study data and reviewed and approved the final manuscript.

## Data Availability

The datasets generated during and/or analysed during the current study are available from the corresponding author on reasonable request.

## References

[1] Sung H (2021). Global cancer statistics 2020: GLOBOCAN estimates of incidence and mortality worldwide for 36 cancers in 185 countries. CA Cancer J. Clin..

[2] Arnold M (2017). Global patterns and trends in colorectal cancer incidence and mortality. Gut.

[3] Li N (2021). Incidence, mortality, survival, risk factor and screening of colorectal cancer: A comparison among China, Europe, and northern America. Cancer Lett..

[4] Kim MK (2014). 2014 clinical practice guidelines for overweight and obesity in Korea. Endocrinol. Metab. (Seoul).

[5] Jee SH (2008). Body mass index and cancer risk in Korean men and women. Int. J. Cancer.

[6] Schlienger JL, Luca F, Vinzio S, Pradignac A (2009). Obesity and cancer. Rev. Med. Interne..

[7] Vecchie A (2018). Obesity phenotypes and their paradoxical association with cardiovascular diseases. Eur. J. Intern. Med..

[8] Christakoudi S (2021). Weight change in middle adulthood and risk of cancer in the European Prospective Investigation into Cancer and Nutrition (EPIC) cohort. Int. J. Cancer.

[9] Jochem C, Leitzmann M (2016). Obesity and colorectal cancer. Recent Results Cancer Res..

[10] Dai Z, Xu YC, Niu L (2007). Obesity and colorectal cancer risk: Aa meta-analysis of cohort studies. World J. Gastroenterol..

[11] Guh DP (2009). The incidence of co-morbidities related to obesity and overweight: A systematic review and meta-analysis. BMC Public Health.

[12] Moghaddam AA, Woodward M, Huxley R (2007). Obesity and risk of colorectal cancer: A meta-analysis of 31 studies with 70,000 events. Cancer Epidemiol. Biomark. Prev..

[13] Campbell PT (2012). Impact of body mass index on survival after colorectal cancer diagnosis: The Cancer Prevention Study-II Nutrition Cohort. J. Clin. Oncol..

[14] Dignam JJ (2006). Body mass index and outcomes in patients who receive adjuvant chemotherapy for colon cancer. J. Natl. Cancer Inst..

[15] Bassett JK (2010). Body size, weight change, and risk of colon cancer. Cancer Epidemiol. Biomark. Prev..

[16] Rapp K (2008). Weight change and cancer risk in a cohort of more than 65,000 adults in Austria. Ann. Oncol..

[17] Thygesen LC (2008). Prospective weight change and colon cancer risk in male US health professionals. Int. J. Cancer.

[18] Laake I (2010). A prospective study of body mass index, weight change, and risk of cancer in the proximal and distal colon. Cancer Epidemiol. Biomark. Prev..

[19] Aleksandrova K (2013). Adult weight change and risk of colorectal cancer in the European Prospective Investigation into Cancer and Nutrition. Eur. J. Cancer.

[20] Steins-Bisschop CN (2014). Weight change later in life and colon and rectal cancer risk in participants in the EPIC-PANACEA study. Am. J. Clin. Nutr..

[21] Samanic C, Chow WH, Gridley G, Jarvholm B, Fraumeni JF (2006). Relation of body mass index to cancer risk in 362,552 Swedish men. Cancer Causes Control.

[22] Chapman IM (2008). Obesity in old age. Front. Horm. Res..

[23] Shaukat A, Dostal A, Menk J, Church TR (2017). BMI is a risk factor for colorectal cancer mortality. Dig. Dis. Sci..

[24] Harriss DJ (2009). Lifestyle factors and colorectal cancer risk (1): Systematic review and meta-analysis of associations with body mass index. Colorectal Dis..

[25] Frezza EE, Wachtel MS, Chiriva-Internati M (2006). Influence of obesity on the risk of developing colon cancer. Gut.

[26] Terry PD, Miller AB, Rohan TE (2002). Obesity and colorectal cancer risk in women. Gut.

[27] Lee J, Lee JS, Park SH, Shin SA, Kim K (2017). Cohort profile: The National Health Insurance Service-National Sample Cohort (NHIS-NSC), South Korea. Int. J. Epidemiol..

[28] Cheol-Seong S (2017). Data resource profile: The national health information database of the national health insurance service in South Korea. Int. J. Epidemiol..

[29] James PT, Leach R, Kalamara E, Shayeghi M (2001). The worldwide obesity epidemic. Obes. Res..

[30] Kwon H, Han KD, Park CY (2019). Weight change is significantly associated with risk of thyroid cancer: A nationwide population-based cohort study. Sci. Rep..

